# Association Between Obesity and Lower Short- and Long-Term Mortality in Coronary Care Unit Patients: A Cohort Study of the MIMIC-III Database

**DOI:** 10.3389/fendo.2022.855650

**Published:** 2022-04-04

**Authors:** Junlue Yan, Xinyuan Li, Wenjie Long, Tianhui Yuan, Shaoxiang Xian

**Affiliations:** ^1^ The First Clinical School, Guangzhou University of Chinese Medicine, Guangzhou, China; ^2^ Department of Community Health, Shenzhen Traditional Chinese Medicine Hospital, Shenzhen, China; ^3^ Geriatrics Department, The First Affiliated Hospital of Guangzhou University of Chinese Medicine, Guangzhou, China; ^4^ Lingnan Medical Research Center, Guangzhou University of Chinese Medicine, Guangzhou, China; ^5^ Cardiovascular Department, The First Affiliated Hospital of Guangzhou University of Chinese Medicine, Guangzhou, China

**Keywords:** obesity paradox, body mass index, mortality, CCU, MIMIC-III database

## Abstract

**Introduction:**

Obesity has long been considered an independent risk factor for cardiovascular diseases (CVD), even in the COVID-19 pandemic. However, recent studies have found that a certain degree of obesity may be beneficial for patients who have already suffered from CVD, which is called the “obesity paradox”. Our objective was to investigate whether the obesity paradox existed in coronary care unit (CCU) patients and the relationship between body mass index (BMI) and short- and long-term mortality.

**Methods:**

We performed a cohort analysis of 3,502 adult CCU patients from the Medical Information Mart for Intensive Care III (MIMIC-III) database. The patients were divided into four groups according to the WHO BMI categories. Both multivariable logistic regression and Cox regression were used to reveal the relation between BMI and mortality. Subgroup analyses were performed based on Simplified Acute Physiology Score (SAPS) and age.

**Results:**

After adjusting for confounders, obese patients had 33% and 30% lower mortality risk at 30-day and 1-year (OR 0.67, 95% CI 0.51 to 0.89; HR 0.70, 95% CI 0.59 to 0.83; respectively) compared with normal-weight patients, while the underweight group were opposite, with 141% and 81% higher in short- and long-term (OR 2.41, 95% CI 1.37 to 4.12; HR 1.81, 95% CI 1.34 to 2.46; respectively). Overweight patients did not have a significant survival advantage at 30-day (OR 0.91, 95% CI 0.70 to 1.17), but did have a 22% lower mortality risk at 1-year (HR 0.78; 95% CI 0.67 to 0.91). The results were consistent after being stratified by SAPS and age.

**Conclusion:**

Our study supports that obesity improved survival at both 30-day and 1-year after CCU admission, and the obesity paradox existed in CCU patients.

## Introduction

According to the World Health Organization (WHO) report, worldwide obesity has nearly tripled since 1975. More than 1.9 billion (39%) adults are overweight, of which over 650 million (13%) are obese (1). With the change of our high-calorie diet habits and sedentary lifestyle, it is easy to predict that the incidence rate of obesity will continue to rise in the near future, imposing a heavy burden upon individuals and societies.

A wealth of epidemiological and clinical evidence has demonstrated a relationship between obesity and a broad spectrum of cardiovascular diseases (CVD), including coronary heart disease (CHD), heart failure (HF), atrial fibrillation (AF), and sudden cardiac death (SCD) (2, 3). It was proved that patients with obesity showed more extensive coronary atherosclerosis and more vulnerable plaque phenotypes (4), which increases the risk for CHD. Data from the Framingham study initially suggested that each increment of one in body mass index (BMI) increased the risk of HF by 5% for men and 7% for women (5). Recently, a study further reflected that obesity was more strongly related to HF with preserved rather than reduced ejection fraction, particularly in women (6). There are also meta-analyses demonstrating an increased risk of AF or SCD with BMI increment (7, 8). Obesity has long been considered an independent risk factor for CVD (9), even in the context of COVID-19 pandemic (10).

However, in recent years, the “obesity paradox” has been debated more frequently, which means that obesity may have a protective effect on patients to some extent, and this phenomenon exists in CVD as well (11–16). Studies have shown that a moderate increase in BMI is associated with significantly lower in-hospital mortality in patients with acute myocardial infarction (AMI) (11, 12), which was consistent with the results of a meta-analysis of 20 prospective studies (13). Meanwhile, mild obesity was verified that independently associated with better activities of daily living and survival in HF patients (14, 15). A meta-analysis including 96424 patients further demonstrated the beneficial effect of appropriate weight gain on survival in HF (16).

Nevertheless, some argued that the obesity paradox in CVD might be a product of biases involving reverse causation and confounding by smoking (17). It was also observed that during long-term follow-up (20 years), the obesity paradox seemed to disappear in patients who had undergone coronary artery bypass grafting (18). A recent retrospective cohort study from China even displayed that the readmission rate of obese patients with AMI increased markedly within one year (19).

Thus, the relationship between obesity and CVD remains controversial and complicated, while the influences of age, comorbidity, and some other aspects on the prognostic role of obesity have yet to be determined. The coronary care unit (CCU) is a well-equipped system for the management of severe CVD. Therefore, we decided to use the data of CCU from the Medical Information Mart for Intensive Care III (MIMIC-III) (20) database to investigate the short- and long-term outcomes of obesity on CVD.

## Materials and Methods

### Data Sources

Data used for analysis was extracted from the MIMIC-III database version 1.4, which is a large, open-access database comprising de-identified health-related data associated with over 40,000 patients who stayed in intensive care units of the Beth Israel Deaconess Medical Center (Boston, MA, USA) between 2001 and 2012 (20). We were obliged to complete the National Institutes of Health’s web-based course and pass the exams to gain access to the database. Informed consent was not required as all the data are anonymous.

### Study Population

**Figure 1:**
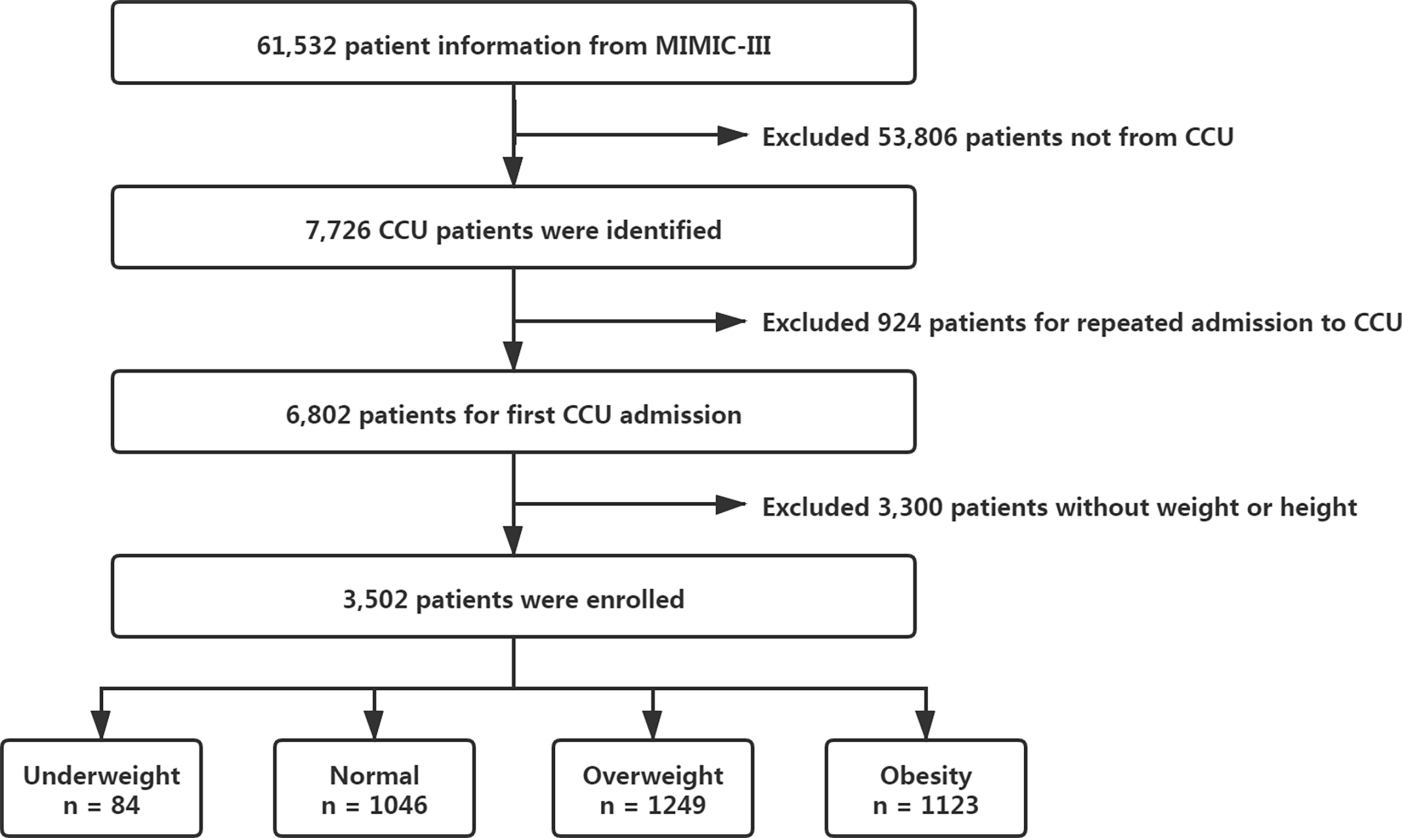
Flowchart of the study.

MIMIC-III contains a total of 61,532 patient information, and we subsequently extracted our dataset about patients first admitted to CCU (**Figure 1**). Anyone lack of weight or height record was excluded. A final of 3502 patients was included for our study (all aged over 18 years). BMI was calculated as (weight (in kilograms)/height (in meters)^2^). We then divided patients into four groups according to the WHO BMI categories: underweight (< 18.5 kg/m^2^), normal weight (18.5 to < 25 kg/m^2^), overweight (25 to < 30 kg/m^2^), and obese (≥ 30 kg/m^2^).

### Data Extraction and Outcomes

We used the Structured Query Language (SQL) in PostgreSQL (v9.6; PostgreSQL Global Development Group) to extract the data, and cords of the SQL were obtained from https://github.com/MIT-LCP/mimic-code/. The following variables were extracted: demographic characteristics (age, gender, ethnicity, marital status), weight, height, admission type, comorbidity, the severity of organ dysfunction (Simplified Acute Physiology Score, SAPS; Sequential Organ Failure Assessment, SOFA), and interventions within the first 24h after CCU admission (renal replacement therapy, mechanical ventilation, and vasopressor).

The primary outcomes were the 30-day and 1-year mortality following CCU admission. The secondary outcomes included hospital mortality and length of stay of CCU or hospital. The mortality numbers are cumulative, which means that the 30-day mortality group includes most of the patients who died in the hospital; however, the few patients that died in the hospital after 30 days were not included in the 30-day mortality.

### Statistical Analysis

Initially, univariate analysis was conducted to compare the study variables between BMI categories. Continuous variables were compared by Kruskal-Wails test across groups and categorical variables by Pearson’s chi-square test or Fisher’s exact test as appropriate.

In addition, smooth curve fitting was used to explore the crude relationship between BMI and short-term mortality. Multivariable logistic regression analysis was performed to predict hospital and 30-day mortality risk by BMI category after adjusting for a set of covariates. Selection for the variables were *P*-value < 0.1 in the univariate test. Multicollinearity for independent variables was assessed by the variance inflation factor (VIF), and no VIF > 4.

We also carried out the log-rank test in the Kaplan-Meter survival analysis to compare the 1-year survival rate between groups and constructed multivariable Cox regression models to estimate the effect of BMI on 1-year all-cause mortality. Loss to follow-up was addressed as censoring values.

Furthermore, we removed the underweight group (with too few statistics but high mortality) and reanalyzed the final models to find out whether the underweight patients distorted the conclusion. Some subgroup analyses were performed based on SAPS and age to determine their confounding influences.

All our data were processed and analyzed by R v4.0.5 (R Foundation for Statistical Computing, Vienna, Austria). A two-sided *P*-value < 0.05 was considered statistically significant.

## Results

### Patient Characteristics

Univariate analyses of demographic and hospitalization characteristics by BMI category were shown respectively in **Tables 1**, **2**. 29.9% patients were in the normal-weight group, 35.7% were overweight, 32.1% were obesity, while only 2.4% were underweight. Overweight and obese patients tended to be younger and married compared with the normal-weight patients. In obese patients, common comorbidities like diabetes and chronic pulmonary disease (COPD) seemed to have a higher incidence rate, but their average SAPS were slightly lower than those of the normal-weight patients.

**Table 1:** Univariate analysis of demographic characteristics by BMI category.

	Total	Underweight <18.5	Normal 18.5 to < 25	Overweight 25 to < 30	Obesity ≥30	*P*value
***n* (%)**	3502	84 (2.4)	1046 (29.9)	1249 (35.7)	1123 (32.1)	
**Male, *n* (%)**	2125 (60.7)	37 (44.1)	560 (53.5)	831 (66.5)	697 (62.1)	<0.001
**Age (years), mean (SD)**	68.2 (14.7)	70.2 (16.9)	71.5 (15.0)	68.8 (14.0)	64.2 (14.0)	<0.001
**Age (years), *n* (%)**						<0.001
** <45**	254 (7.3)	9 (10.7)	59 (5.6)	78 (6.2)	108 (9.6)	<0.001
** 45-65**	1097 (31.3)	17 (20.2)	258 (24.7)	374 (29.9)	448 (39.9)	<0.001
** 65-80**	1294 (37.0)	28 (33.3)	353 (33.8)	498 (39.9)	415 (37.0)	0.022
** 80+**	857 (24.5)	30 (35.7)	376 (36.0)	299 (23.9)	152 (13.5)	<0.001
**Race, *n* (%)**						0.68
** White**	2469 (70.5)	62 (73.8)	737 (70.5)	877 (70.2)	793 (70.6)	0.92
** Black**	222 (6.3)	7 (8.3)	60 (5.7)	76 (6.1)	79 (7.0)	0.52
** Other/Unknown**	811 (23.2)	15 (17.9)	249 (23.8)	296 (23.7)	251 (22.4)	0.53
**Marital status, *n* (%)**						<0.001
** Married**	1864 (53.2)	29 (34.5)	526 (50.3)	702 (56.2)	607 (54.1)	<0.001
** Single/Divorced/** ** Separated/Unknown**	1029 (29.4)	32 (38.1)	292 (27.9)	350 (28.0)	355 (31.6)	0.047
** Widowed**	609 (17.4)	23 (27.4)	228 (21.8)	197 (15.8)	161 (14.3)	<0.001

**Table 2:** Univariate analysis of hospitalization characteristics by BMI category.

	Total	Underweight <18.5	Normal 18.5 to < 25	Overweight 25 to < 30	Obesity ≥30	*P*value
**Admission type, *n* (%)**						0.17
** Elective**	179 (5.1)	2 (2.4)	66 (6.3)	57 (4.6)	54 (4.8)	
** Emergency/Urgent**	3323 (94.9)	82 (97.6)	980 (93.7)	1192 (95.4)	1069 (95.2)	
**Comorbidity, *n* (%)**						
** Congestive heart failure**	409 (11.7)	13 (15.5)	122 (11.7)	141 (11.3)	133 (11.8)	0.71
** Cardiac arrhythmias**	381 (10.9)	9 (10.7)	123 (11.8)	132 (10.6)	117 (10.4)	0.75
** Valvular disease**	143 (4.1)	3 (3.6)	51 (4.9)	50 (4)	39 (3.5)	0.43
** Hypertension**	517 (14.8)	11 (13.1)	162 (15.5)	173 (13.9)	171 (15.2)	0.65
** Diabetes**	1101 (31.4)	14 (16.7)	244 (23.3)	371 (29.7)	472 (42.0)	<0.001
** Chronic pulmonary disease**	670 (19.1)	31 (36.9)	197 (18.8)	221 (17.7)	221 (19.7)	<0.001
** Renal failure**	635 (18.1)	13 (15.5)	203 (19.4)	215 (17.2)	204 (18.2)	0.52
** Liver disease**	123 (3.5)	1 (1.2)	45 (4.3)	32 (2.6)	45 (4.0)	0.060
** Metastatic cancer**	72 (2.1)	4 (4.8)	27 (2.6)	30 (2.4)	11 (1.0)	0.004
**Severity of organ dysfunction, mean (SD)**						
** SAPS**	17.5 (5.5)	18.4 (4.7)	17.9 (5.3)	17.4 (5.7)	17.1 (5.5)	<0.001
** SOFA**	4.0 (3.1)	4.0 (3.1)	4.0 (3.0)	3.9 (3.1)	3.9 (3.2)	0.35
**CCU interventions in the first 24h, *n* (%)**						
** Renal replacement therapy**	128 (3.7)	2 (2.4)	44 (4.2)	42 (3.4)	40 (3.6)	0.71
** Mechanical ventilation**	1112 (31.8)	27 (32.1)	313 (29.9)	402 (32.2)	370 (33.0)	0.48
** Vasopressor**	1225 (35.0)	29 (34.5)	372 (35.6)	443 (35.5)	381 (33.9)	0.84
**Length of stay, median (Q1-Q3)**						
** CCU LOS**	2.8 (1.5-5.5)	2.6 (1.3-4.6)	2.9 (1.6-5.2)	2.8 (1.5-5.4)	2.8 (1.5-5.9)	0.96
** Hospital LOS**	6.6 (3.6-11.7)	6.1 (3.5-11.4)	6.7 (3.8-12.0)	6.5 (3.5-11.4)	6.6 (3.5-11.5)	0.75
**Mortality, *n* (%)**						
** Hospital mortality**	465 (13.3)	21 (25)	158 (15.1)	165 (13.2)	121 (10.8)	<0.001
** 30-day mortality**	542 (15.5)	27 (32.1)	189 (18.1)	194 (15.5)	132 (11.8)	<0.001
** 1-year mortality**	1013 (28.9)	48 (57.1)	373 (35.7)	342 (27.4)	250 (22.3)	<0.001

SAPS, Simplified Acute Physiology Score; SOFA, Sequential Organ Failure Assessment; CCU, coronary care unit; LOS, length of stay.

It was worth noting that although no significant trend was found for either CCU or hospital length of stay across BMI categories, the crude hospital, 30-day, and 1-year mortality rates did decline with the increase of BMI. In general, some differences existed between the BMI groups with respect to various covariates, but none of the differences seem to be significant enough to account for the effect of BMI on mortality.

### BMI and Short-Term Mortality

**Figure 2:**
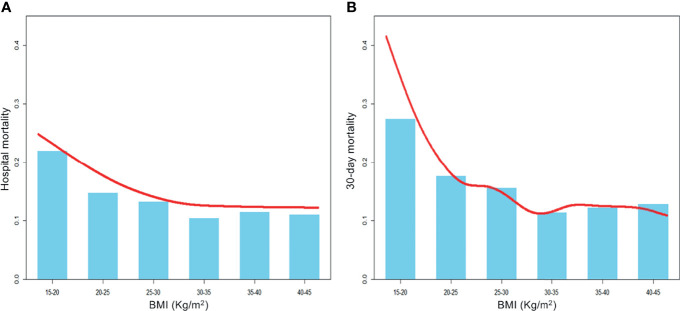
A smooth curve fitting for the relationship between BMI and the risk of hospital **(A)** and 30-day **(B)** mortality. It is observed that BMI was negatively correlated with short-term mortality before any adjustments.

Taking BMI as a continuous variable, we visually explored the crude relationship between BMI and hospital or 30-day mortality by using the spline smoothing fitting, which was presented in **Figure 2**. It was clear that BMI was negatively correlated with short-term mortality.

Before adjusting for any covariates, we estimated crude hospital and 30-day mortality risk by BMI category through the univariate logistic regression models. The results have shown that obese patients had significantly lower mortality risk in both hospital (OR 0.68, 95% CI 0.53 to 0.87) and 30-day (OR 0.60, 95% CI 0.47 to 0.77) compared with normal-weight patients. Contrarily, underweight patients had markedly higher risk in both periods (hospital, OR 1.87, 95% CI 1.09 to 3.11; 30-day, OR 2.15, 95% CI 1.31 to 3.45).

Relations between BMI and short-term mortality that were revealed by our multivariable regression models were very similar to those of the univariate regression analyses (**Table 3**). Even after adjusting the clinical confounders listed, the multivariable model did not appreciably change the results that the univariate risk estimated: the obese group had mortality risks that were 28% lower in hospital (OR 0.72, 95% CI 0.54 to 0.98) and an additional 5% lower at 30-day (OR 0.67, 95% CI 0.51 to 0.89) compared with the normal weight group, while underweight patients had 110% and 141% higher mortality risk in two time periods, respectively (hospital, OR 2.10, 95% CI 1.31 to 3.77; 30-day, OR 2.41, 95% CI 1.37 to 4.12).

**Table 3:** Multivariable logistic regression results for hospital and 30-day mortality.

	Hospital Odds ratio (95% CI)	30-day Odds ratio (95% CI)
**BMI category (ref, Normal (18.5 to < 25))**		
** Underweight (<18.5)**	2.10 (1.13-3.77)^a^	2.41 (1.37-4.12)^b^
** Overweight (25 to < 30)**	0.90 (0.68-1.19)	0.91 (0.70-1.17)
** Obesity (≥30)**	0.72 (0.54-0.98)^a^	0.67 (0.51-0.89)^b^
**Male (ref, Female)**	0.92 (0.72-1.19)	0.83 (0.66-1.05)
**Age category (ref, < 45 years)**		
** 45 to < 65 years**	1.35 (0.74-2.61)	1.15 (0.66-2.11)
** 65 to < 80 years**	1.49 (0.82-2.86)	1.36 (0.78-2.17)
** 80+ years**	2.13 (1.15-4.17)^a^	2.22 (1.26-4.11)^b^
**Race (ref, White)**		
** Black**	0.68 (0.39-1.13)	0.69 (0.42-1.12)
** Other/Unknown**	1.43 (1.10-1.86)^b^	1.39 (1.09-1.77)^b^
**Marital status (ref, Married)**		
** Single/Divorced/Separated/Unknown**	1.39 (1.07-1.81)^a^	1.21 (0.94-1.55)
** Widowed**	1.03 (0.73-1.43)	0.96 (0.70-1.30)
**Admission type (ref, Elective)**		
** Emergency/Urgent**	4.42 (1.97-12.04)^b^	3.36 (1.70-7.53)^b^
**Comorbidity**		
** Congestive heart failure**	1.45 (1.01-2.07)^a^	1.29 (0.92-1.81)
** Cardiac arrhythmias**	1.12 (0.77-1.61)	1.40 (1.00-1.96)
** Valvular disease**	0.77 (0.45-1.27)	0.78 (0.48-1.26)
** Hypertension**	d	0.71 (0.44-1.18)
** Diabetes**	d	d
** Chronic pulmonary disease**	1.31 (1.00-1.72)^a^	1.34 (1.04-1.72)^a^
** Renal failure**	0.80 (0.59-1.09)	1.09 (0.68-1.71)
** Liver disease**	2.28 (1.36-3.76)^b^	1.51 (0.90-2.49)
** Metastatic cancer**	3.26 (1.69-6.07)^c^	3.86 (2.13-6.84)^c^
**Severity of organ dysfunction**		
** SAPS**	1.15 (1.12-1.19)^c^	1.13 (1.10-1.17)^c^
** SOFA**	1.17 (1.11-1.23)^c^	1.18 (1.12-1.24)^c^
**CCU interventions in the first 24h**		
** Renal replacement therapy**	0.76 (0.44-1.28)	0.82 (0.49-1.35)
** Mechanical ventilation**	1.05 (0.78-1.42)	0.94 (0.71-1.25)
** Vasopressor**	1.24 (0.94-1.64)	1.14 (0.88-1.47)

^a^P < 0.05; ^b^P < 0.01; ^c^P < 0.001; ^d^Dropped out of the final multivariable model.

As expected, older age, emergency admission, complicated with COPD or metastatic cancer, and higher SAPS or SOFA score were associated with increased short-term mortality risk (*P* < 0.05). Racial minorities seemed to have a higher risk of mortality; however, the other ethnic group contained a large number of patients who were unable to obtain ethnic identity. These patients might be more critical and have a worse prognosis, which could lead to biased results. Interestingly, patients with diabetes dropped out of the final regression models for both hospital and 30-day mortality risk.

### BMI and Long-Term Mortality

**Figure 3:**
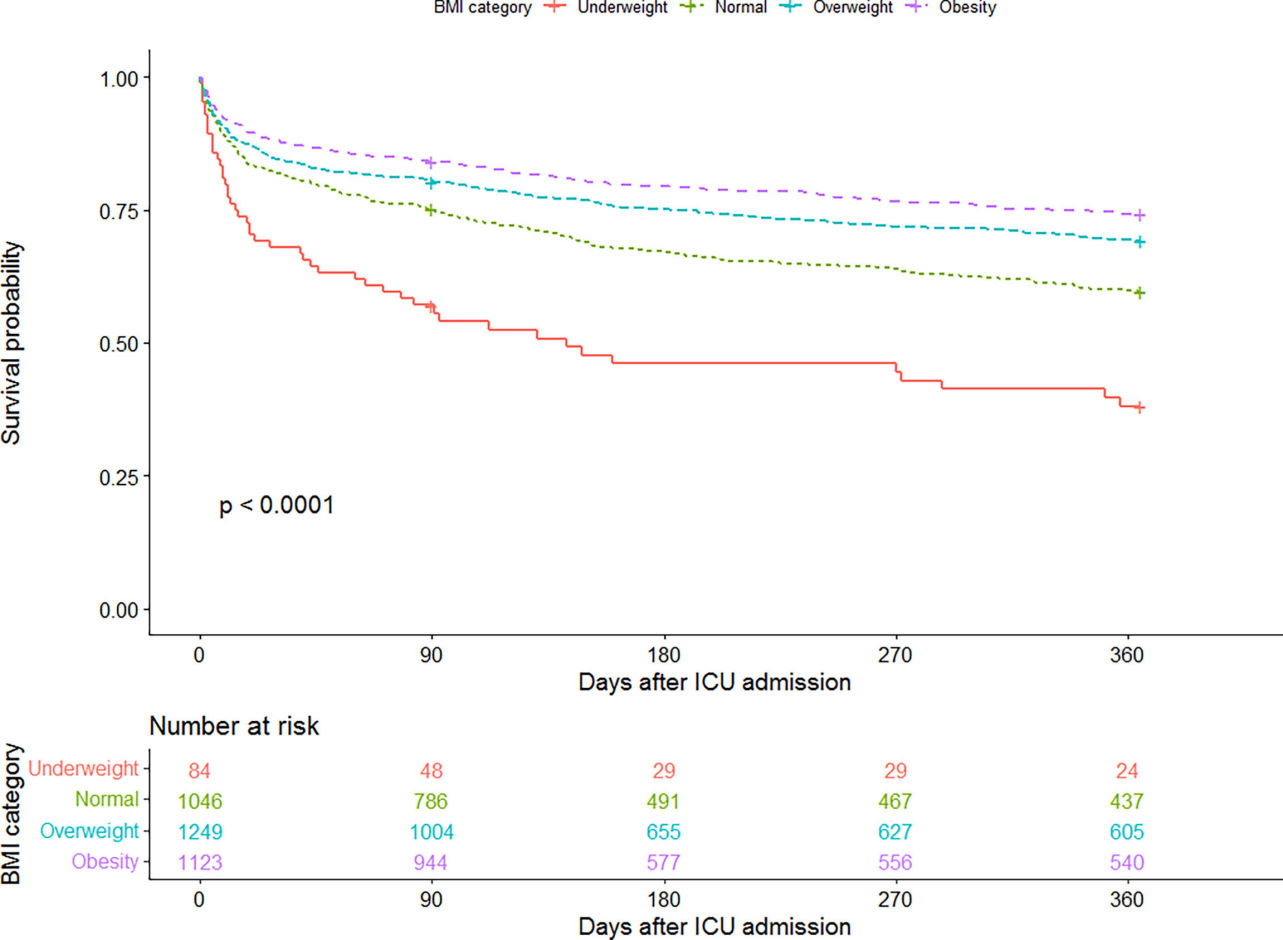
Kaplan-Meier curve for 1-year survival by BMI category. The number at risk represents the patients’ number in each BMI category at the start of each time period.

Kaplan-Meier survival curves at 1-year were shown in **Figure 3**, indicating the notable survival advantage for overweight and obese patients compared with their normal-weight counterparts over time (log-rank test, *P* < 0.001).

Our multivariable Cox regression analysis further demonstrated the strong relationship between BMI and long-term survival (**Table 4**). In the crude model without any adjustments, overweight and obese patients had markedly lower risks of 1-year mortality (HR 0.73, 95% CI 0.63 to 0.85; HR 0.59, 95% CI 0.50 to 0.69; respectively) than patients with normal weight, but underweight patients took a significantly higher risk (HR 1.87, 95% CI 1.39 to 2.53). After adjusting for all potential confounders, the association remained independent (underweight, HR 1.81, 95% CI 1.34 to 2.46; overweight, HR 0.78, 95% CI 0.67 to 0.91; obese, HR 0.70, 95% CI 0.59 to 0.83; **Table 4**, model 2).

**Table 4:** Multivariable Cox regression analysis for 1-year mortality.

1-year mortality (ref, Normal (18.5 to < 25))	Non-adjusted Hazard ratio (95% CI)*P* value	Adjust I Hazard ratio (95% CI)*P* value	Adjust II Hazard ratio (95% CI)*P* value
**Underweight (<18.5)**	1.87 (1.39-2.53) <0.001	1.85 (1.37-2.50) <0.001	1.81 (1.34-2.46) <0.001
**Overweight (25 to < 30)**	0.73 (0.63-0.85) <0.001	0.81 (0.70-0.94) 0.004	0.78 (0.67-0.91) 0.001
**Obesity (≥30)**	0.59 (0.50-0.69) <0.001	0.73 (0.62-0.87) <0.001	0.70 (0.59-0.83) <0.001

Adjusted I for gender, age, race and marital status;

Adjusted II for gender, age, race, marital status, admission type, congestive heart failure, cardiac arrhythmias, valvular disease, hypertension, diabetes, chronic pulmonary disease, renal failure, liver disease, metastatic cancer, SAPS, SOFA, renal replacement therapy, mechanical ventilation and vasopressor.

### Sensitivity and Subgroup Analyses

After the removal of underweight patients, we retained 3418 subjects, and analyses without the underweight group did not change the primary results for the rest of BMI categories, regardless of short- or long-term. Besides, removing the underweight group would not reduce the mortality advantage of patients with increased BMI compared with those with normal.

**Figure 4:**
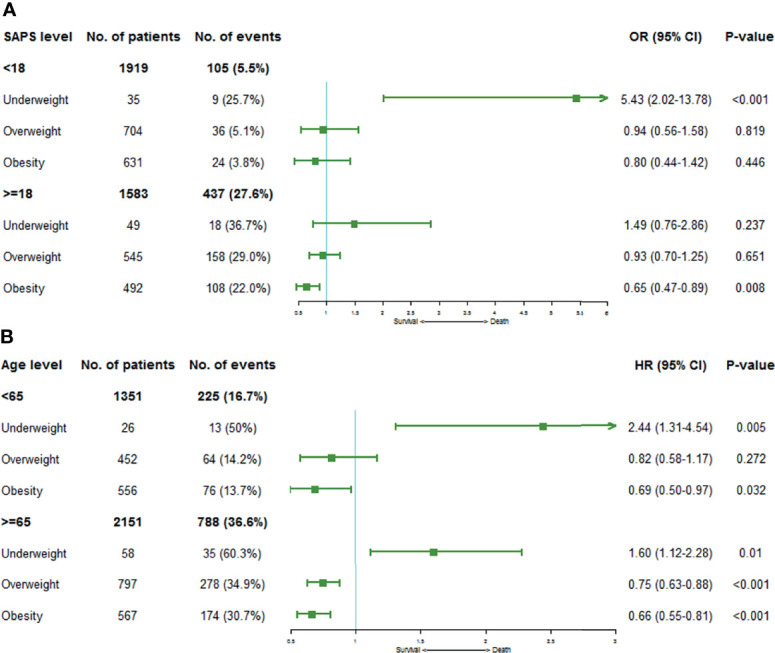
Forest plots depicting the ORs or HRs of mortality risks on the stratification of SAPS and age levels by BMI category. **(A)** shows ORs of 30-day mortality stratified by SAPS levels, and **(B)** demonstrates HRs of 1-year mortality on the stratification of age levels. Squares represent ORs or HRs with 95% CIs demonstrated as horizontal bars. Normal weight category acts as the reference group.

To further explore the relation between BMI and 30-day or 1-year mortality in different stratifications, we performed the subgroup analyses based on SAPS and age, respectively (**Figure 4**). It was clear that even after careful adjustments, the increase of BMI was still associated with the decrease of short- and long-term mortality.

## Discussion

In our research, CCU patients who were obese had markedly lower 30-day and 1-year mortality risks despite having higher incidences of diabetes or COPD compared with their normal-weight counterparts. Such protective effect of obesity has also been reported in other studies using the same MIMIC-III database but with different populations, including sepsis (21), acute kidney injury (22), and acute respiratory distress syndrome (23).

One reason for the obesity paradox in CVD may relate to the inaccuracy of BMI in characterizing the extent of obesity (24, 25). Although BMI is commonly the standard for classifying weight, there are some pitfalls in BMI as it does not distinguish between lean mass and fat mass (26). People with higher BMI may not have increased fat mass necessarily, like athletes (27), while normal BMI does not preclude an individual from owning more fat mass and thus increases the risk of CVD (28). Particularly, the increased lean mass in obese patients probably plays an essential role as it has been associated with better long-term outcomes (29, 30). A retrospective study of over 47,000 patients with HF disclosed a significant protective effect of lean mass at the aspect of all-cause mortality, regardless of BMI or fat mass (31). Therefore, to further untangle the obesity paradox in CVD, assessment of comprehensive body composition compartments, rather than BMI alone, may be more suitable for the evaluation of obesity.

Another explanation focuses on the complex effects of adipose tissue. On the one hand, it is recognized that adipose tissue distribution influences the prognosis of CVD, where visceral adiposity leads to deleterious metabolic disturbances, but subcutaneous fat accumulation has a benign effect on cardiac metabolism (32, 33). Research in CHD patients manifested that compared with visceral adipose tissue, subcutaneous fat had lower infiltration by M1 macrophages (pro-inflammatory) and expressed cardioprotective adipokines (e.g., adiponectin) at a higher level (34). On the other hand, most of the patients admitted to CCU are in the critical stage, and adipose tissue undergoing such circumstances will further accumulate alternatively activated M2 macrophages, which have local anti-inflammatory and insulin-sensitizing properties (35). During the prolonged phase of a critical period, adipose tissue has also been validated to increase the expression of adiponectin as well as anti-inflammatory cytokines, show a better capacity to store glucose and lipid metabolites, and thereby reducing their potential toxicity (36). All these above may contribute to the prognosis of obese patients with CVD.

The protection provided by increased nutritional reserves may benefit the mortality as well (37, 38). The early phases of critical disease are characterized by an increase in energy expenditure, which results in a catabolic state (39). While obese patients are able to provide more substrate synthesis energy to meet the higher needs during such a period due to their high lipid storage (40). On the contrary, a study including 1,000 chronic HF patients, which assessed their weight at the first visit, 1-year follow-up, and vital status after 3 years, finally showed that weight loss was associated with higher long-term mortality (41). Another recent research of 1,515 HF patients with preserved ejection fraction reflected the same conclusion (42).

Other potential mechanisms include lower activation of the sympathetic nervous system (43) and higher circulating progenitor cell (CPC) counts (44) in obesity patients. It has been proved that persisting activation of the sympathetic nervous system will lead to poor prognosis of HF (45). Nevertheless, compared with the lean hypertensive patients, the obese showed lesser increases in renin and epinephrine (46). Such attenuated neurohormonal responses to stress may help them relieve the toxic effects of sympathetic activation. CPCs are mononuclear cells that related to an endogenous regenerative or reparative system, contributing to vascular repair as well as regeneration (47). While obesity was independently associated with higher CPC counts, which may indicate a better prognosis (44).

What’s more, clinicians tend to consider obese patients at higher risk of worse outcomes, thereby resulting in more attention and more active use of prophylactic measures (48, 49). Others hypothesized that the increased nursing needs of obese patients might lead to a lower threshold for intensive care unit admission, thus some fared better because they were initially less sick (50, 51), just as the younger obese patients in our study. The feature that obese patients are younger also existed in the earlier large cohort study using the MIMIC-II database (52). However, after we controlled for all confounders in our multivariable models, the survival advantage of obesity persisted, so did the earlier study (52). We even further carried out stratified analysis based on SAPS and age level, and the results were still consistent.

There are several limitations within our research. First, as a retrospective single-center study, it has the same common problem as other observational studies did, which is that the influence of some potential confounding factors is hard to be completely excluded. Second, we used height and weight recorded at CCU admission to calculate BMI, but due to the nature of the MIMIC-III database, we were unable to determine whether the patient received fluid resuscitation prior to CCU admission. Third, in order to ensure the accuracy and integrity of the data, we directly removed patients without weight or height records, which led to a considerable part of data loss. Finally, the obesity paradox in CCU patients presented by our study does not imply causality but just associations, the molecular and physiological mechanisms behind it need to further expound and prove.

## Conclusions

Our investigation verified the existence of the obesity paradox once again. For CCU patients, the benefit of obesity on improving prognosis is significant, regardless of short or long term. The mechanisms behind this relationship may be complicated and need to be further explained.

## Data Availability Statement

The original contributions presented in the study are included in the article/supplementary material. Further inquiries can be directed to the corresponding authors.

## Ethics Statement

Ethical review and approval were not required for the study on human participants in accordance with the local legislation and institutional requirements. Written informed consent for participation was not required for this study in accordance with the national legislation and the institutional requirements.

## Author Contributions

JY participated in the research design, data collecting, and writing of the paper. XL participated in the data analysis. WL participated in the data cleaning and revising of the paper. TY and SX provided substantial advice in designing the study and assisting in writing and revising the paper. All authors contributed to the article and approved the submitted version.

## Funding

This work was supported by: (1) Youth Talent Promotion Project of China Association of Chinese Medicine (2020-2022) (No.2020-QNRC2-09), (2) National Key Research and Development Program of China (No.2017YFC1700304), (3) National Key Research and Development Program of China (No. 2018YFC1707401), (4) National Clinical Research Base of Traditional Chinese Medicine (No. [2018]131).

## Conflict of Interest

The authors declare that the research was conducted in the absence of any commercial or financial relationships that could be construed as a potential conflict of interest.

## Publisher’s Note

All claims expressed in this article are solely those of the authors and do not necessarily represent those of their affiliated organizations, or those of the publisher, the editors and the reviewers. Any product that may be evaluated in this article, or claim that may be made by its manufacturer, is not guaranteed or endorsed by the publisher.
